# *In vivo* methods and applications of xenon-129 magnetic resonance

**DOI:** 10.1016/j.pnmrs.2020.11.002

**Published:** 2021-02

**Authors:** Helen Marshall, Neil J. Stewart, Ho-Fung Chan, Madhwesha Rao, Graham Norquay, Jim M. Wild

**Affiliations:** POLARIS, Imaging Sciences, Department of Infection, Immunity and Cardiovascular Disease, University of Sheffield, Sheffield, United Kingdom

**Keywords:** Hyperpolarised ^129^Xe, Magnetic resonance imaging/spectroscopy, Lungs, Brain, Kidneys

## Abstract

Hyperpolarised gas lung MRI using xenon-129 can provide detailed 3D images of the ventilated lung airspaces, and can be applied to quantify lung microstructure and detailed aspects of lung function such as gas exchange. It is sensitive to functional and structural changes in early lung disease and can be used in longitudinal studies of disease progression and therapy response. The ability of ^129^Xe to dissolve into the blood stream and its chemical shift sensitivity to its local environment allow monitoring of gas exchange in the lungs, perfusion of the brain and kidneys, and blood oxygenation. This article reviews the methods and applications of *in vivo*^129^Xe MR in humans, with a focus on the physics of polarisation by optical pumping, radiofrequency coil and pulse sequence design, and the *in vivo* applications of ^129^Xe MRI and MRS to examine lung ventilation, microstructure and gas exchange, blood oxygenation, and perfusion of the brain and kidneys.

## Highlights

• Review of *in vivo* methods and applications of ^129^Xe magnetic resonance in humans.
• Focus on polarisation physics, radiofrequency coil and pulse sequence design.
• ^129^Xe MRS/MRI is sensitive to lung ventilation, microstructure and gas exchange.
• ^129^Xe lung MR can detect early disease, disease progression and therapy response.
• Dissolved ^129^Xe MR can monitor blood oxygenation, lung, brain and kidney perfusion.

## Graphical abstract

**Figure:**
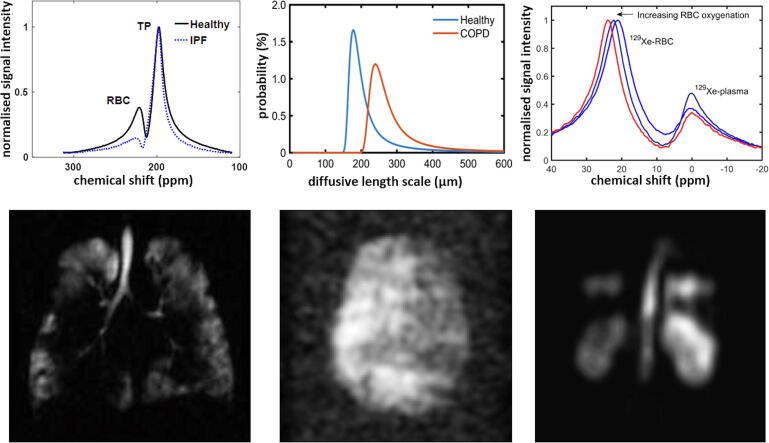

## 1 Introduction

The noble gas xenon was discovered in 1898 by William Ramsay and Morris Travers, and named from the Greek for “stranger” [1]. Of the nine naturally occurring isotopes of xenon, only ^131^Xe and ^129^Xe have non-zero spin, which permits magnetic resonance. ^131^Xe has spin 3/2 and a nuclear electric quadrupole moment (which dominates spin-lattice relaxation, shortening T_1_ to milliseconds), while ^129^Xe has spin 1/2, no quadrupole moment and a gyromagnetic ratio 3.4 times larger than ^131^Xe [2]. Xenon is an excellent probe of its chemical environment because it is inert and monatomic with a large spherical electron cloud, the distortion of which affects the NMR chemical shift [3], and ^129^Xe MR spectroscopy has been employed as such in many studies to determine the properties of a diverse range of microporous solids [2], [3].

Laser optical pumping, where circularly polarised light of a suitable wavelength is used to drive the electron spins of certain atoms into non-Boltzmann energy level distributions, was discovered by Kastler in 1950 [4]. In 1960, Bouchiat et al*.* from the same institute, ENS in Paris, showed that angular momentum could be transferred from the electron spins of optically pumped rubidium vapour to the nuclear spins of ^3^He gas [5], the first demonstration of spin-exchange optical pumping (SEOP, see Section 2). The first application of SEOP to ^129^Xe was demonstrated in 1978 [6] and the technique was further developed by the Happer group at Princeton [7], [8], which eventually led to hyperpolarised ^129^Xe NMR spectroscopy [9] and the first biomedical imaging studies in the 1990s. In 1994 Albert et al*.* presented the first MR images of hyperpolarised ^129^Xe in excised mouse lungs [10]. The potential of MR imaging of ^129^Xe in both its gas-phase and dissolved-phase in the lungs, and subsequent uptake in to the circulatory system, brain and other organs was recognised in the first publications on the subject [10], [11], [12]. The first *in vivo* hyperpolarised gas MR images of human lungs were demonstrated by Ebert et al*.* [13] and MacFall et al*.* [14] in 1996 using ^3^He, and Mugler et al*.* in 1997 using ^129^Xe, along with ^129^Xe spectra of the chest and head [11]. We now perform ^129^Xe MRI routinely in patients for clinical investigations of lung disease [15].

The noble gases ^3^He and ^129^Xe are particularly suitable as inhaled contrast agents for MR imaging. Noble gases are characteristically safe, non-toxic and unreactive. The ^3^He and ^129^Xe isotopes have a nuclear spin of 1/2, yielding a two-state nuclear energy level structure in the presence of a magnetic field. Crucially, their nuclear polarisations can be dramatically increased using SEOP, facilitating MR signal enhancements of up to 4–5 orders of magnitude. The gyromagnetic ratios of ^3^He and ^129^Xe, pertaining to the available MR signal, are roughly 75% and 25% that of ^1^H respectively (Table 1). Helium is almost completely insoluble in the lung parenchyma [16] and blood [17] (Ostwald solubility coefficient < 0.01) due to its tightly bound electron cloud. In contrast, the large, polarisable electron cloud of xenon allows it to dissolve in parenchymal tissue, blood plasma and red blood cells (Ostwald solubility coefficients are: ~0.1 [18], ~0.09 [19] and ~0.2 [19] respectively). It is this non-negligible solubility that permits uptake of xenon into the bloodstream and the subsequent inhibition of N-methyl-D-aspartate receptors in the neuronal cells that is believed to lead to the anaesthetic function of xenon gas [20]. Despite this effect, MR imaging of inhaled ^129^Xe is safe and well-tolerated in healthy volunteers and patients with pulmonary disease [21], [22], including children as young as 6 years old [23]; the ^129^Xe dose is 1 L or less and breath-holds are short (usually < 16 s) and thus the alveolar concentration is well below the minimum alveolar concentration required to induce anaesthesia. Since the xenon electron cloud is easily distorted, the local magnetic field at the site of the nucleus is readily altered in different chemical environments. As a result, ^129^Xe displays a broad range of NMR chemical shift values; of the order of hundreds of ppm when dissolved in various liquids and biological tissues [24].

**Table 1:** Properties of ^1^H, ^3^He and ^129^Xe.

Properties	^1^H	^3^He	^129^Xe
Isotope abundance (%)^a^	99.99	1.37 × 10^−4^	26.44
Nuclear spin^a^	½	½	½
Gyromagnetic ratio (MHz/T)^a^	42.58	−32.43	−11.78
Spin density (10^19^ atoms/cm^3^)^b^	6690	2.37	2.37
Chemical shift range (ppm)^c^	~10	~0.8	~250
Self-diffusion coefficient (cm^2^/s)^d^, ^e^	2 × 10^−5^	2.05	0.062
Free diffusion coefficient (in air) (cm^2^/s)^e^	–	0.86	0.14
Ostwald solubility in water^f^	–	0.0096–0.0101	0.083–0.093
Ostwald solubility in blood^f^	–	0.008–0.0104	0.137–0.222

a From Ref. [300].

b From Ref. [301], ^1^H spin density in water, gas spin densities at 1 atm and 37 °C.

c From Refs. [24], [302], chemical shift offset from gaseous phase resonance (0 ppm).

d From Ref. [303], self-diffusion coefficient of water at 25 °C.

e From Ref. [176], assuming gas at 1 atm and 37 °C, and an air mixture of 79% N_2_ and 21% O_2_.

f From Ref. [18], for gas at 1 atm and 37 °C.

Due to the higher gyromagnetic ratio and relative ease of polarisation [12], ^3^He has historically been the focus of hyperpolarised gas lung MRI research until recently [25], [26]. However, the ^3^He isotope is rare (being only produced during tritium decay in the nuclear industry) and in recent years has become increasingly scarce and expensive [21], [27]. Xenon occurs naturally in the atmosphere in small concentrations (87 ppb), 26.44% of which is constituted by the ^129^Xe isotope. After extraction from the air via liquefaction and gas separation, xenon gas can be isotopically enriched to increase the ^129^Xe fraction up to 80–90%. The availability of ^129^Xe, coupled with progress in ^129^Xe polarisation and imaging technology, has motivated a shift towards ^129^Xe for imaging the airspaces of the lungs, and renewed interest in utilising ^129^Xe for the investigation of gas exchange, lung perfusion, blood oxygenation, and gas uptake in the brain and kidneys.

Hundreds of millions of people worldwide suffer from chronic respiratory diseases including asthma, chronic obstructive pulmonary disease (COPD), cystic fibrosis (CF) and interstitial lung disease (ILD) [28]. Pulmonary function tests are the clinical standard for assessment of lung disease but they only provide global metrics about the function of the lungs as a whole, and are insensitive to early-stage lung disease and subtle changes in lung function. Sensitive, repeatable measures that provide regional information about lung structure and function are essential for the early detection and thorough assessment of spatially heterogeneous lung diseases. High resolution computed tomography (CT) is the gold standard for structural imaging of the lungs; however, it does not provide functional information and entails a significant radiation dose [29], which is a particular problem in high risk patient groups such as pregnant women and children, and in diseases such as CF where longitudinal monitoring is necessary [30]. Ventilation-perfusion scintigraphy and single photon emission tomography (SPECT) are used clinically to image lung ventilation and perfusion function, yet they suffer from poor spatial resolution and the risks of ionising radiation.

Proton (^1^H) MRI of the lungs is limited by the low proton density of the lung parenchyma and signal loss due to the magnetic susceptibility differences between the numerous air-tissue interfaces (short T_2_* [31]), with respiratory and cardiac motion presenting additional challenges [32], [33]. Advances in scanner hardware and pulse sequence design have enabled the assessment of lung morphology (structure) with ^1^H MRI [32], for example using ultra-short echo-time (UTE) sequences [34], [35], which can provide an alternative to CT in some situations [35], [36]. Functional lung images can be obtained with ^1^H MRI [37] either by using external contrast agents which alter T_1_ relaxation times, as with dynamic contrast enhanced perfusion imaging [38] and oxygen-enhanced imaging [39], or by using the modulation of the proton signal caused by respiratory and cardiac motion to infer lung ventilation and perfusion indirectly [40]. While these techniques offer the advantage of functional imaging without the need for additional hardware, hyperpolarised gas MRI allows direct imaging of the inhaled contrast agent and can be tuned to different aspects of lung function, and also probe more distal organs in the case of hyperpolarised ^129^Xe MRI.

This review will focus on the methodology and applications of hyperpolarised ^129^Xe MRI and MRS, covering: 1. optical pumping physics, 2. imaging physics considerations, 3. radiofrequency coils, 4. ventilation imaging, 5. diffusion-weighted imaging and modelling, 6. dissolved-phase ^129^Xe lung MRI and MRS, 7. ^129^Xe dissolved in human blood, and 8. imaging inhaled ^129^Xe beyond the lungs.

## 2 Spin-exchange optical pumping physics

While it is possible to perform MR imaging on thermally polarized (i.e. non-hyperpolarised) ^129^Xe (for example in high-pressure cells for the purpose of quality control testing), the low density of gases compared to protons in water and biological tissue renders the NMR signal of this non-hyperpolarised ^129^Xe too low for *in vivo* lung imaging in practice. To overcome this inherent sensitivity limitation, the nuclear spin polarisation of ^129^Xe can be enhanced beyond its thermal equilibrium Boltzmann polarisation, resulting in a “hyperpolarised” (HP) ^129^Xe nuclear spin system.

**Fig. 1:**
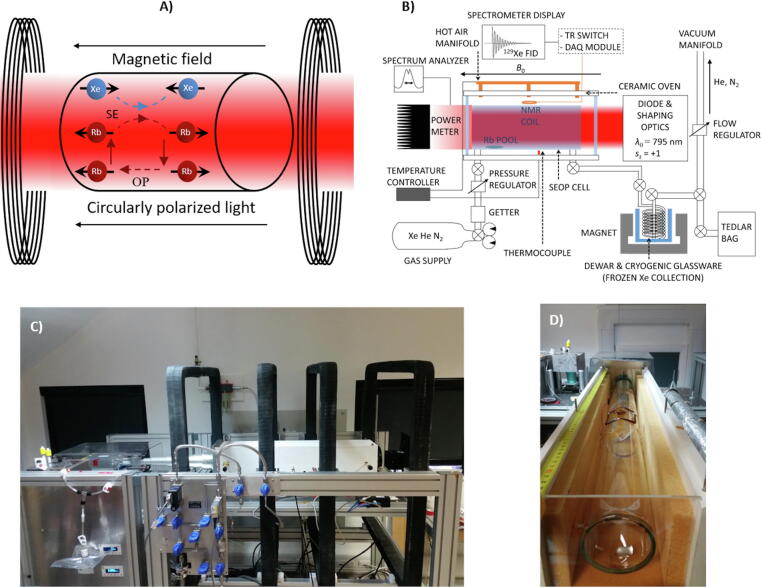
Optical pumping. (a) Simplified conceptual picture of Rb-^129^Xe spin-exchange optical pumping (SEOP). SEOP is a two-step process involving (i) the spin polarisation of Rb valence electrons through optical pumping of Rb vapour with circularly polarised light resonant with the Rb *D*_1_ transition and (ii) transfer of the Rb electron polarisation to the nuclei of ^129^Xe via spin-exchange collisions. The black arrows depict the polarisation of the Rb electron and ^129^Xe nuclear spin states. OP and SE represent optical pumping and spin-exchange interactions, respectively. (b) Schematic of the key functional components of a continuous-flow Rb-^129^Xe polariser, adapted with permission from [59]. A stopped-flow set-up is essentially the same without the need for a cryostat and magnet (see bottom right). (c) Photo of ^129^Xe spin-exchange optical pumping polariser located at the POLARIS laboratory, University of Sheffield. (d) Ceramic oven containing *V* = 3530 mL SEOP (7.5 cm diameter, 80 cm length) cell without the lid. The pool of rubidium is visible at the cell gas entrance (bottom of photo). Incident laser light P = 150 W (BrightLock 200 W, QPC, CA, USA), 794.77 nm wavelength. Gas mixture throughout: 3% Xe, 87% He, 10% N_2_.

Although HP ^129^Xe can be produced by dynamic nuclear polarisation [41], the technique most commonly used to hyperpolarise ^129^Xe nuclei for MR applications is rubidium (Rb) spin-exchange optical pumping (SEOP) [6], [8]. SEOP is a two-step physical process involving (i) polarisation of the valence electrons in Rb vapor through absorption of circularly polarised light (optical pumping) and (ii) collisional energy transfer from the polarised Rb electrons to ^129^Xe nuclei (spin exchange) – see Fig. 1. SEOP with ^129^Xe generally requires relatively low densities of xenon gas (0.01–0.25 xenon partial pressure) owing to high destruction rates of Rb electron polarisation at elevated xenon concentrations [42]. The gas mixture used for ^129^Xe-SEOP is therefore diluted with a buffer gas – typically either a ^4^He-N_2_ mixture or pure N_2_ gas – which also serves to (i) prevent emission of non-circularly polarised photons during electronic Rb relaxation [43]^1^; and (ii) pressure broaden the Rb D_1_ linewidth, which improves photon absorption efficiency [44].

Typically, one of two approaches to produce HP ^129^Xe with SEOP is used: the “stopped- flow (SF)” (also known as “batch”) mode [45], [46], [47], [309], in which higher-density xenon gas mixtures (up to 25% xenon concentration) are dispensed directly from the SEOP cell; and (ii) the “continuous-flow (CF)” mode [48], [49], [50], [51], [52], [53], [54], in which a lower-density xenon gas mixture (1–3% xenon concentrations) is allowed to flow through the SEOP cell over a period of time, and xenon is cryogenically separated from the buffer gases. While higher ^129^Xe polarisation values have been reported in SF-SEOP [46], [47], [55], [56], the xenon production rates are generally of the order of 100 mL/h, with the highest observed being ~1000 mL/h [56], which can be compared to >1000 mL/h characteristic of CF-SEOP [48], [49], [51]. There is therefore a trade-off between the achievable ^129^Xe polarisation and the xenon volume production rate when considering SF- and CF-SEOP methods for a given application. For example, for MR applications of HP ^129^Xe in a clinical setting, CF-SEOP is most suitable, as it is critical to have a large volume production rate in order to enable large volumes of ^129^Xe to be produced on demand; whereas for applications where high-throughput and large volumes of xenon are not required (e.g. *in vitro* NMR ^129^Xe applications), SF-SEOP is more suitable.

A useful metric to evaluate ^129^Xe polariser performance is the dose equivalent rate, DE_rate_ = *fQ*_Xe_*P*_Xe_, which expresses the xenon volume production rate (*Q*_Xe_) of 100% polarised ^129^Xe and 100% isotopically enriched xenon, where *f* is the isotopic fraction of ^129^Xe and *P*_Xe_ is the ^129^Xe nuclear spin polarisation [58]. Through employment and optimization of a large SEOP cell (3530 mL volume), a high HP ^129^Xe production efficiency on a continuous-flow polariser has recently been reported [59], with a DE_rate_ of 1013 mL/h. This has enabled routine clinical lung MRI with hyperpolarised ^129^Xe doses available on demand at run time as well as high signal-to-noise ratio (SNR) ^129^Xe MRI of the human brain [60], [61], [62] and kidneys [63].

## 3 Imaging physics considerations

The induced hyperpolarisation of ^129^Xe is not renewable; that is, the polarisation relaxes by longitudinal (T_1_) relaxation to the equilibrium (Boltzmann) polarisation, which is a factor of 10^4^-10^5^ times lower than the induced hyperpolarisation. This decay is accelerated when applying radiofrequency (RF) pulses. Thus, the longitudinal magnetisation of ^129^Xe decays continuously from its maximum, *M_0_*, according to RF excitation and T_1_, described by the following equation, assuming a constant flip angle, *θ*, and gradient spoiling between RF pulses [64]:

(1) $$M_{n}=M_{0}exp\left(-\frac{\left(n-1\right)TR}{T_{1}}\right)\cos^{n-1}\left(\theta \right)$$

where *M_n_* is the longitudinal ^129^Xe magnetisation after *n* RF pulses and TR is the inter-pulse repetition time.

The intrinsic gaseous T_1_(^129^Xe) is of the order of hours at room temperature, measured at a Xe density of 0.15 amagat [65]. However, the decay of hyperpolarised ^129^Xe signal in the lungs is orders of magnitude more rapid (T_1_(^129^Xe) approximately 20 s [66]), primarily due to the presence of paramagnetic oxygen. Intermolecular dipolar coupling between molecular oxygen and the ^129^Xe nuclei leads to a linear dependence of the longitudinal relaxation rate 1/T_1_(^129^Xe) of the gas on the partial pressure of oxygen (pO_2_) [67]. The T_2_*(^129^Xe) value in the alveoli is considerably longer than lung T_2_*(^1^H) values (approximately 1.4 ms at 1.5 T [31]), and is influenced by lung inflation level (T_2_*(^129^Xe) = 25 ms at FRC + 1 L and 52 ms at TLC at 1.5 T) as well as B_0_ strength (18 ms at FRC + 1 L and 24 ms at TLC at 3 T) [68]. As the acquired MR signal is boosted by hyperpolarisation of the gas, image SNR is largely independent of B_0_ at the current clinically-used magnetic fields (1.5 T and 3 T) [68]; however, increased T_2_*(^129^Xe) dephasing in regions of B_0_ field inhomogeneity such as near blood vessels and the diaphragm is evident at 3 T.

To conserve the non-renewable hyperpolarised gas signal as much as possible, low-flip angle gradient echo and steady-state free precession sequences form the foundation of hyperpolarised gas pulse sequence design [64]. As the available magnetisation is partly depolarised by each RF pulse, RF depolarisation filters are imposed in *k*-space such that an image phase encoded with sequential ordering in Cartesian space has lower SNR but higher spatial frequency detail than an otherwise identical acquisition using centric Cartesian phase encoding [69]. Variable flip-angle schemes have been proposed to counteract this effect [64], [70], [71], where the flip angle is stepwise increased during image acquisition to maintain constant transverse magnetisation, but in practice these schemes can be difficult to implement in a robust fashion due to B_1_ inhomogeneity [72] and RF power limitations considering the low gyromagnetic ratio of ^129^Xe. Non-Cartesian *k*-space acquisition trajectories lead to different RF *k*-space depolarisation filters, causing artefacts and loss of spatial resolution, which can be compensated for after signal acquisition or mitigated by using non-sequential acquisition order to distribute the effects of the filter more evenly over *k*-space [73], [74].

In 2D hyperpolarised gas imaging, the distribution of effective flip angles over a realistic selected slice thickness causes the uniformity of the slice profile to decrease with increasing phase encoding number [69], [72]. The smaller flip angles experienced at the edges of the slice profile deplete the longitudinal magnetisation of ^129^Xe gas at a slower rate than in the slice centre, meaning that the later phase encodes are more heavily weighted by signal from the slice edges. In addition, diffusion of “fresh” hyperpolarised gas from outside the selected slice that has not been affected by the RF pulses adds to the in-slice signal throughout the acquisition. These effects can cause errors in slice-selective techniques which use temporal signal decay to calculate parameters such as the partial pressure of oxygen in the lung (pO_2_) [75].

Diffusion of hyperpolarised gas within the lung airspaces during imaging limits the achievable spatial resolution and leads to signal attenuation resulting from the imaging gradients, reducing the effective transverse relaxation time, though this effect is minor compared to RF depolarisation [69] and less pronounced for ^129^Xe compared to ^3^He due to its lower diffusion coefficient. The confining structure of healthy alveoli limits the distance that gas molecules can travel but in some diseases such as emphysema the alveolar walls are damaged or destroyed, allowing increased diffusion and therefore exacerbating related effects [76]. However, the sensitivity of hyperpolarised gas diffusion to its surrounding structural environment can be exploited as a unique means to obtain information about lung microstructure, as addressed in Section 6.

### 3.1 Pulse sequences

Whilst 2D spoiled gradient echo (SPGR) scans acquired with contiguous slices spanning the whole of the lungs are robust, signal-to-noise benefits can be obtained with balanced steady-state sequences that exploit the long T_2_ of hyperpolarised gases by recycling the transverse magnetisation remaining at the end of each repetition time [77], [78]. However, these sequences are sensitive to off-resonance effects, which can lead to banding artefacts in regions of poor B_0_ homogeneity (e.g. close to the diaphragm); these effects are more pronounced at higher field strengths [78]. SNR gains may also be realised by using 3D gradient echo acquisitions in place of slice-selective 2D equivalents [79], though increased sensitivity to motion can cause image blurring.

Non-Cartesian *k*-space acquisition schemes such as radial [80] and spiral [81], [82] enable time-resolved imaging of ^129^Xe gas with high temporal resolution, to investigate ventilation dynamics. Spiral trajectories can achieve high temporal resolution by an optimal *k*-space encoding efficiency, whilst the temporal resolution of radial encoding is maximised by sharing radial views in a sliding window reconstruction. In addition, centre-out (ultra-short echo time (UTE)) radial and spiral trajectories sample the centre of *k*-space (k_0_, which is representative of the total magnetisation in the imaging volume) every TR. Thus, the time-dependence of the magnitude of the k_0_ point is intrinsically sensitive to magnetisation dynamics; this idea can be used to correct hyperpolarised ^129^Xe images for the filtering effects of RF-induced depolarisation [74]. This approach was recently extended to allow regional mapping of the RF depolarisation (and, therefore, of the flip angles) by using a keyhole reconstruction technique, wherein radial datasets are divided into two temporally resolved “keys” post-acquisition [83]. However, despite the advantages for monitoring of ^129^Xe magnetisation dynamics, the SNR of 3D radial ^129^Xe MR images has been reported to be lower than a dose equivalent 2D multi-slice SPGR sequence [58]. Novel trajectories for hyperpolarised ^129^Xe MRI, including spiral-based techniques for efficient encoding of 3D *k*-space such as Fermat Looped ORthogonally Encoded Trajectories (FLORET) [84], are a subject of keen interest. 3D radial UTE sequences show good promise for imaging of pulmonary gas exchange with ^129^Xe MRI, where short T_2_*(^129^Xe) is a limiting factor (as discussed in 3.3, 7).

### 3.2 Acceleration techniques

Due to the time-dependence of the magnetisation, MRI with hyperpolarised gas is particularly suitable for image acceleration techniques which reduce the number of RF pulses required to acquire an image, such as parallel imaging [85], [86] and compressed sensing [87]. As fewer RF pulses are required, higher flip angles can be used to acquire data with increased signal [88], [89], [90]. Thus, for hyperpolarised gases, there is no SNR penalty proportional to 1/√R as is observed in thermally-polarised parallel imaging (where R is the “acceleration” factor by which the number of phase encoding steps is reduced compared to full Fourier encoding) [86], enabling high acceleration factors with little degradation of the image quality [90], [91], [92], [93]. Compressed sensing has the advantage that it does not require multiple receiver coils and has been successfully implemented to accelerate ^129^Xe imaging and enable: acquisition of ^129^Xe images and ^1^H anatomical MR images in a single breath-hold which aids their registration [94]; high-resolution multiple *b*-value diffusion-weighted imaging [95], [96]; increased temporal resolution in dynamic ^129^Xe imaging [94], [97]; and combined acquisition of diffusion-weighted and gas-exchange ^129^Xe imaging in a single breath-hold [98]. Furthermore, prior knowledge can be used to improve image reconstruction and achieve higher acceleration, for instance by using knowledge of the magnetisation decay or structural information from ^1^H MR images [99], or the sparsity of complex difference images for gas flow applications [100]. As hyperpolarised gas MR images are inherently sparse, it is possible to simplify the reconstruction process by skipping the sparsifying transformation step in some cases [101].

### 3.3 Additional considerations for MR imaging of ^129^Xe in the “dissolved phase”

Upon inhalation, xenon partially dissolves in the lung parenchymal tissue and blood plasma (Ostwald solubility coefficient ~0.1) and red blood cells (0.27) [102]. In the lungs, there is a distinct downfield chemical shift of ^129^Xe dissolved in the lung parenchymal tissue and blood plasma (referred to as TP or “barrier” by some research groups) of 197 ppm and in the red blood cells (RBCs) of 216–222 ppm, (with respect to the resonance of gaseous-phase ^129^Xe in the alveolar airspace) – see Fig. 4b and Table 2. By acquiring MR signals on a timescale similar to that during which ^129^Xe exchanges between these compartments, the chemical shift phenomenon can be exploited to quantitatively assess pulmonary gas exchange function. This so-called “dissolved-phase” ^129^Xe gas exchange MRS/MRI is a subject of active research (see Section 7).

**Table 2:** ^129^Xe chemical shifts in somatic substances.

Solvent	Chemical Shift (ppm)
Distilled water	190^a^
Saline (0.9% NaCl)	194^b^
Olive oil	198^c^
Rat adipose tissue	191^d^
Blood plasma	194^a^ 197^e^
Erythrocytes (red blood cells)	216^a^ 222^e^
Perfluorooctyl bromide emulsion (PFOB)	106^f^

The offset is expressed in parts per million (ppm) from the gaseous-phase ^129^Xe resonance (0 ppm).

a From Ref. [304].

b From Ref. [305].

c From Ref. [24].

d From Ref. [220].

e From Ref. [223].

f From Ref. [306], PFOB is a blood substitute.

Dissolved-phase ^129^Xe MRS/MRI presents several additional challenges when compared to conventional hyperpolarised MRI of alveolar ^129^Xe gas. Firstly, the complex magnetic environment of the lungs and large susceptibility gradients between airspace and tissue lead to extremely short transverse relaxation times of ^129^Xe dissolved in the lung parenchyma and blood (T_2_*(^129^Xe) ≲ 2 ms). Therefore, dissolved-phase ^129^Xe signal is usually acquired as a free induction decay or echo with very short echo time. In light of the benefit of longer T_2_*(^129^Xe) values [103], considerable development work for dissolved ^129^Xe MRI/MRS in humans has been performed at a field strength of 1.5 T (T_2_*(^129^Xe) ~2.2 ms [104]), and this has recently been shown to be translatable to 3 T (T_2_*(^129^Xe) ~1.1 ms) [105]. In addition, upon diffusing into the lung parenchyma, ^129^Xe rapidly exchanges between the parenchyma and pulmonary capillaries (typical time constant for airspace to capillary transfer <100 ms [106], [107]), and within the capillaries, ^129^Xe exchanges between plasma and RBCs on a timescale of 12 ms [108]. Dissolved phase ^129^Xe MRI/MRS is further complicated by the low signal (~2% of that of the gaseous phase ^129^Xe), which arises from the low tissue/gas volume ratio (~0.2) and low solubility of xenon in parenchymal tissue (0.1). Fortunately, the gaseous phase ^129^Xe acts as a reservoir to replenish dissolved-phase magnetisation after its depolarisation and thus a relatively high flip angle can be applied to the dissolved phase. This necessitates careful design of RF excitation pulses to selectively excite the dissolved phase ^129^Xe whilst minimising off-resonant excitation of the gaseous phase reservoir [109], [110]. Despite these challenges, the longitudinal relaxation time of ^129^Xe gas in the alveolar spaces (T_1_(^129^Xe) ~ 25 s at 1.5 T [68]) is longer than that of a typical breath-hold scan duration, and in blood (T_1_(^129^Xe) ~ 6–13 s [111], [112], [113]) is sufficient to enable detection in distal organs such as the brain [114], [115] and kidneys [63] (see Section 9).

## 4 Radio frequency coils

The low gyromagnetic ratio (−11.78 MHz T^−1^), unit dielectric constant (1.00126) and nonconductive properties of gaseous ^129^Xe predominantly define the design considerations for radio frequency (RF) coils [116]. Due to the low Larmor frequency of ^129^Xe (17.66 MHz at 1.5 T and 35.33 MHz at 3.0 T) when compared to ^1^H (63.83 MHz at 1.5 T and 127.6 MHz at 3.0 T), the Ohmic loss of the RF coil is lower, as it is proportional to the square root of the resonant frequency (√ω) [117]. Radiation loss is negligible as the dimensions of the RF coil are very small when compared to the Larmor wavelength, diminishing the likelihood of magnetic dipole radiation. Dielectric losses due to capacitive coupling and dissipation due to inductive coupling with ^129^Xe as an NMR sample in situ in a RF coil are nil due to the near unity dielectric constant and the lack of conductivity [118], [119], [120], [121], [122], and thus, ^129^Xe does not contribute to sample-dominated losses [123]. Hence, the efficiency of RF transmission and detection for ^129^Xe is mainly determined by the filling factor [124] of the RF coil. However, tissues in organs containing ^129^Xe such as the lungs, brain and kidneys do contribute to the sample-dominated loss by inductive coupling, which is proportional to the square of the resonant frequency (ω^2^) [120], [121], [122]. The achievable sample loss, measured as a ratio ($Q_{\mathrm{Coil}}=Q_{\mathrm{Loaded}}∙Q_{\mathrm{Unloaded}}^{-1}$) of the quality factor with ($Q_{\mathrm{Loaded}}$) and without ($Q_{\mathrm{Unloaded}}$) the sample in situ [123], which measures coupling of an RF coil to the sample, is proportional to ${R\omega }^{\frac{3}{2}}+1$ [120], where ω is the resonant frequency and R is a constant that depends on inductive coupling and conductive losses. Although $Q_{\mathrm{Coil}}$ does not provide a direct measurement of SNR, by estimating the inductive coupling of the RF coil with the sample, it provides an indication of the sensitivity of the RF coil to the anatomy or airspace containing ^129^Xe. Thus, in order to optimise a ^129^Xe RF coil for efficiency and SNR, both the filling factor [124] that confirms sensitivity towards the sample space and sample losses that confirm sample domination [123], [125], [126] should be optimised. In contrast, for conventional RF coils for proton imaging, optimising for sample-loss implicitly optimises filling factor as the same sample both induces losses and generates NMR signal.

As the Larmor frequency of ^129^Xe is much lower than that of ^1^H, receive-only RF coil arrays with a large number of channels, which are adapted to the subject with close proximity, provide relatively moderate improvements in SNR over transceiver RF coils [92] compared to those seen for ^1^H receiver RF coil arrays [127], [128]. Thus, high density receiver RF coil arrays for ^129^Xe [93] primarily serve the purpose of accelerating imaging by benefitting from the fact that for hyperpolarised gases, the SNR can be preserved when under-sampling *k*-space while increasing the flip angle, which is made possible by the reduced number of RF pulses [91], [92], [93], [129]. A recent approach to increase the SNR of a receiver RF coil array for ^129^Xe was to optimise the filling factor and reduce conductive loss by using superior grade copper [60].

Transmit RF coils for ^129^Xe lung MRI of birdcage topology are large enough to fit an adult human torso and typically have an elliptical or oval shape in the transverse plane orthogonal to$B_{0}$ conforming to the patient table and magnet [93], [130], [131]. The design of a non-cylindrical birdcage is determined by conformal mapping and modal mesh currents [130], [131]. $B_{1}^{+}$ homogeneity (i.e. standard deviation of $B_{1}^{+}$ field amplitude) of 7% is achievable, which may be compared to 16% achieved with a flexible dual Helmholtz coil [130]. However, the latter has advantages in terms of comfort and ease of patients’ entry into the magnet. In contrast, transmit RF coils of birdcage topology for ^129^Xe brain MR imaging have a cylindrical shape with $B_{1}^{+}$homogeneity of 6.5% [60], [114].

In clinical practice, it is often necessary to acquire spatially concordant ^1^H images along with the ^129^Xe images, for planning, structure-function assessment and to arrive at quantitative measures such as ventilation defect percentage [132], [133], [134], [135], [136]. For ^129^Xe brain MRI, complementary ^1^H images of the brain can be acquired in a separate session with separate RF coils, and since the human head is rigid, the images can be easily co-registered without image manipulation [60], [114]. However, in order to co-register ^1^H and ^129^Xe images of the lungs, it is essential to acquire both image sets in the same lung inflation state, and preferably back-to-back in the same breath-hold, ^129^Xe imaging followed by ^1^H imaging [94], [135], [136], [137], [138]. To achieve this the RF coil(s) can be enabled for multi-nuclear lung imaging [139] using multi-tuned RF coils [140], [141], [142], [143], multiple electrically-isolated RF coils [136], [144], [145] or switchable-resonance RF coils [146], [147], or otherwise the scanner’s body ^1^H RF coil can be used along with a dedicated RF coil for ^129^Xe which is electrically isolated for ^1^H [132], [133], [134], [135], [136], [137].

## 5 Ventilation imaging

MR imaging of gaseous-phase HP ^129^Xe during a static breath-hold can provide detailed 3D images of the ventilated airspaces of the lungs. Ventilation function can be quantified using several approaches, which typically require the acquisition of co-registered ^1^H anatomical images along with ^129^Xe ventilation images in order to derive the total lung volume. The most commonly used metric of ventilation is the ventilation defect percentage (VDP) – the percentage of the lung volume with low or no signal on ^129^Xe ventilation MRI, and its counterpart ventilation volume percentage (VV% = 100 – VDP) [133], [148], [149], [150], [151], [152], [153], [154]. ^129^Xe VDP is reproducible [155], [156], [157], [158], increases with age [149], [151], [155] and correlates with pulmonary function tests [155], [156], [157], [159]. Regional ^129^Xe ventilation metrics have shown significant correlation with ventilation/perfusion SPECT and CT percentage emphysema [160], and CT-based surrogates of lung ventilation [161]. In addition, ventilation image signal intensity can be classified into clusters of graded ventilation [148], [150], [151], [154] and its regional coefficient of variation can be calculated to assess ventilation heterogeneity [162], [163].

**Fig. 2:**
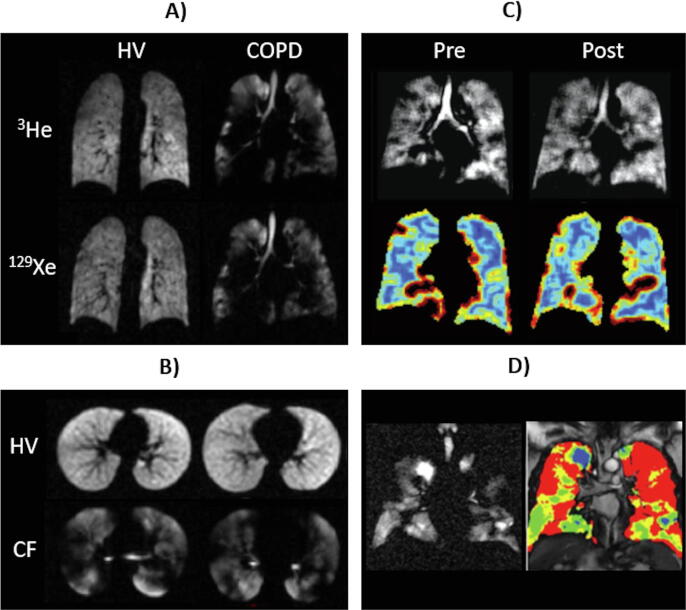
Ventilation imaging. (a) ^3^He and ^129^Xe ventilation images of a healthy non-smoker and a patient with COPD, adapted with permission from [157]. (b) ^129^Xe ventilation images of a healthy 6-year-old (HV, FEV_1_ = 95%) and an 11-year-old with CF (CF, FEV_1_ = 102%), adapted with permission from [165]. (c) ^129^Xe ventilation images (top) and coefficient of variation maps (bottom; blue = low COV, red = high COV) of a patient with asthma pre- and post-bronchodilator inhalation, adapted with permission from [163]. (d) ^129^Xe ventilation image (left) and binning map (right; red = defect, yellow = low intensity, green = medium intensity, blue = high intensity) from an older patient with asthma (FEV_1_ = 53%), adapted with permission from [155]. (In this case, ventilation defect percentage (VDP) is defined as the ratio of the number of red pixels to the total number of pixels in the whole lung × 100).

^129^Xe ventilation imaging is extremely sensitive to obstructive lung disease,^2^ exhibiting increased ventilation heterogeneity and VDP in patients with chronic obstructive lung disease (COPD) [157], [159], asthma [155], [163], [164], [311], cystic fibrosis (CF) [156], [165], [166], [167] and non-small-cell lung cancer [157], compared to healthy volunteers (Fig. 2). Studies in children and young adults with CF [156], [165], [167], lymphangioleiomyomatosis [168] and following allogenic haematopoietic stem cell transplantation [169], where ventilation defects were detected in patients with clinically-normal spirometry (i.e. indicating that ^129^Xe ventilation MRI is sensitive to “sub-clinical” disease), highlight the sensitivity of ^129^Xe ventilation imaging to early-stage lung disease, which could allow earlier interventions to impede disease progression. In children and young adults with CF, ventilation defects are often not associated with structural abnormalities evident on ^1^H ultra-short echo time MRI [167], suggesting that early functional deficits may be detected prior to structural damage.

In addition to early sensitivity, the non-ionising nature of ^129^Xe ventilation MRI, coupled with its safety and tolerability in adults and children [21], [22], [23], make it well suited for longitudinal follow-up post-intervention scans. ^129^Xe ventilation MRI response to therapy has been demonstrated in: patients with asthma after bronchodilator inhalation [163] and bronchial thermoplasty [312], children with CF after pulmonary exacerbation and subsequent treatment with intravenous antibiotics [170], patients with CF after acute maximal exercise [313], patients with bronchial stenosis after airway stent placement [171], and a patient with adenocarcinoma after radiotherapy [172]. In a study monitoring response to antibiotics in children with CF, ^129^Xe VDP showed the largest relative improvement of all outcome measures [170], and ^129^Xe ventilation MRI has been shown to be a sensitive method to assess longitudinal lung disease in children and adults with CF [158], [314]. Increased ^129^Xe ventilation heterogeneity at low lung inflation levels, consistent with airway closure, has been noted in healthy elite divers who were able to exhale beyond the residual volume of the lungs [173], and in healthy volunteers [174], emphasising the need to control for lung inflation level to ensure reliable longitudinal ventilation imaging.

^129^Xe and ^3^He ventilation images of the same patient are visually similar [157], [159], [161] (Fig. 2a), although greater ventilation abnormalities (and quantitatively higher VDP) are often evident on ^129^Xe images when compared to ^3^He images [157], [159], [163], [175], likely due to the lower diffusion coefficient of ^129^Xe in air [176]. Similarly, the lower diffusion coefficient of ^129^Xe in air limits the sensitivity of time-resolved ^129^Xe ventilation imaging for the detection of delayed and collateral ventilation observed in patients with severe COPD using ^3^He MRI [177], [178]. While ^1^H anatomical imaging is often performed in a separate breath-hold, advances in multi-nuclear hardware and compressed sensing have made imaging ^129^Xe and ^1^H within the same breath-hold possible [94], [136], [138]. This provides spatially and temporally registered images of complementary lung ventilation and structure.

Rapid time-resolved ^129^Xe imaging allows visualisation of ventilation and measurement of spatially resolved signal-time curves during a breathing cycle [82], [97], and velocity mapping of gas flow in the major airways [100]. ^129^Xe multiple-breath washout imaging, where dynamic ventilation imaging is performed over several breathing cycles provides spatially resolved information about gas washout complementary to the clinical multi-breath washout pulmonary function test. This provides fully quantitative imaging of fractional ventilation (the turnover of gas per breath), and has been demonstrated in healthy adults [179] and a 9-year-old child with CF [158].

## 6 Diffusion weighted imaging and modelling

The relatively high diffusivity of hyperpolarised noble gas isotopes (Table 1), in comparison to protons in water molecules, is ideally suited for pulsed gradient echo diffusion-weighted MRI, where the random Brownian motion of inhaled ^129^Xe or ^3^He gas atoms in the acinar airspace is used to probe the underlying acinar microstructure. The free diffusion coefficient ($D_{0}$) of ^129^Xe in air (at atmospheric pressure and body temperature) is 0.14 cm^2^/s [176], and over a time-scale of several milliseconds (Δ), ^129^Xe gas atoms diffuse an average distance ($\bar{x}$) of ~0.5 mm as defined by the 1D diffusion equation, $\bar{x}=\sqrt{2D_{0}\Delta }$. The alveoli are the smallest restricting structure of the lungs and have a diameter of ~0.2 mm [180]. Thus, on a time-scale of a few ms, ^129^Xe atoms can encounter the alveolar walls multiple times, restricting the diffusion and leading to a decrease of MR signal attenuation (S) that is described by:

(2) $$S=S_{0}e^{-bD}$$

where $S_{0}$ is the MR signal in the absence of diffusion and the *b*-value (b) represents the diffusion-weighting applied by magnetic field gradients. In the presence of restricting boundaries, the diffusion coefficient (*D*) is called “apparent diffusion coefficient” (ADC), and is sensitive to the underlying alveolar dimensions.

The most commonly employed method for hyperpolarised gas diffusion-weighted MRI is a modified Stejskal and Tanner pulsed gradient echo sequence [181] that uses two bipolar trapezoid gradient pulses of equal area after RF pulse excitation. The diffusion time (Δ) is defined as the interval between the middle of the first gradient lobe and the middle of the second. Hyperpolarised gas diffusion-weighted MRI sequences are typically implemented in an interleaved fashion, where acquisition of each line of *k*-space is repeated for each *b*-value, by altering the diffusion gradient amplitude, before proceeding to the next line. This ensures that each interleaved scan has the same TE and TR, and that motion artefacts and signal attenuation associated with the depolarisation by RF and T_1_ are minimised.

**Fig. 3:**
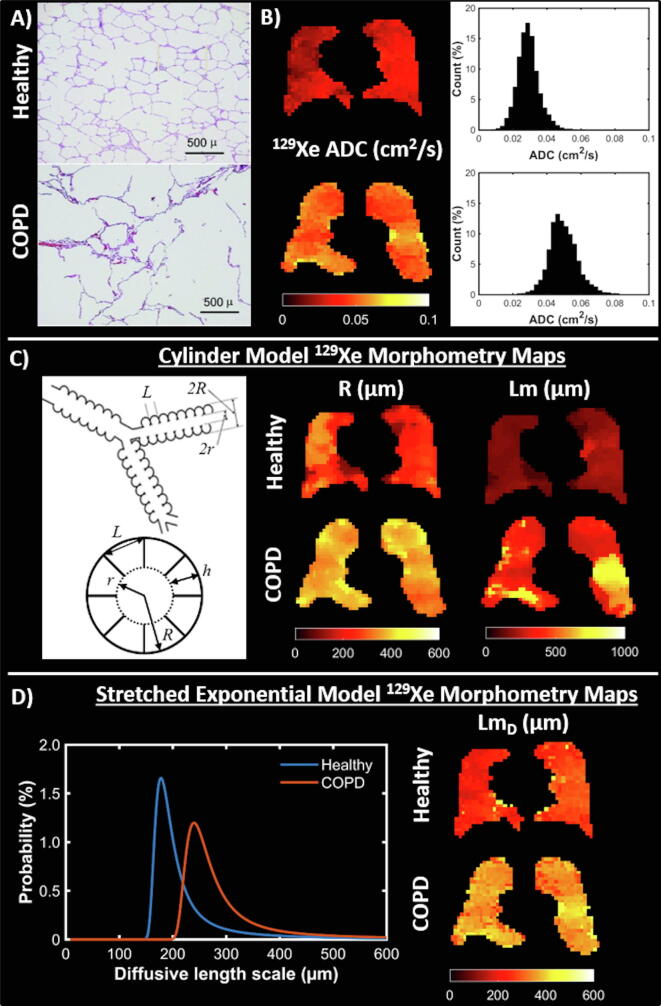
Diffusion imaging. (a) Examples of histological slides from a healthy lung (top) and COPD lung with emphysema (bottom) that are used to calculate mean linear intercept (Lm) measurements, adapted with permission from [307]. (b) ^129^Xe ADC maps and whole lung ADC histograms for a healthy volunteer (23-year-old female, top) and COPD patient (68-year-old male, bottom). (c) (Left) Schematic drawing of the cylindrical model of acinar airway geometry based upon the Haefeli-Bleuer and Weibel acinar geometry [212], adapted with permission from [308]. (c) (Right) Cylinder model ^129^Xe lung morphometry maps of acinar airway radius (R) and mean linear intercept (Lm) in the same healthy volunteer and COPD patient as in (b). (d) (Left) Probability distributions of diffusive length scale derived from the stretched exponential model for the same healthy volunteer and COPD patient. (d) (Right) Stretched exponential model ^129^Xe lung morphometry maps of mean diffusive length scale (Lm_D_) for the same healthy volunteer and COPD patient.

The first *in vivo* human ^129^Xe diffusion measurements were acquired at 1.5 T in two healthy volunteers [182], where a mean ^129^Xe ADC of 0.040 cm^2^/s was obtained using two *b*-values of 0 and 10 s/cm^2^. In subsequent studies of healthy lungs across different field strengths, similar mean ^129^Xe ADC values were obtained, ranging from 0.035 to 0.050 cm^2^/s [157], [159], [183], [184], [185] (Fig. 3b). Healthy lung ^129^Xe ADC values are therefore approximately 3–4 times smaller than the free diffusion coefficient of ^129^Xe in air, and 5–6 times smaller than the respective ^3^He ADC values (~0.2 cm^2^/s) in healthy lungs [186], [187], reflecting the inherently lower diffusivity of the ^129^Xe gas.

**Fig. 4:**
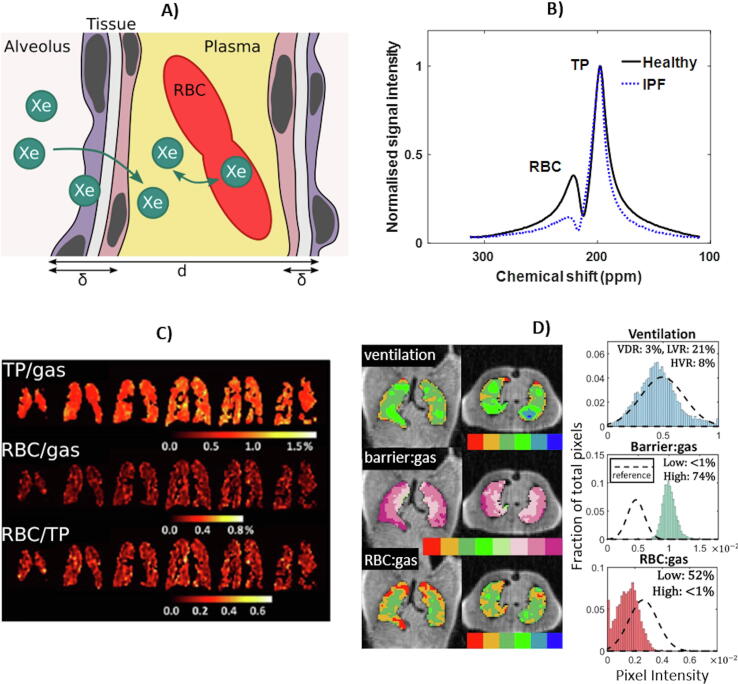
Probing gas exchange. (a) Cartoon of diffusive exchange of xenon gas from alveolus to capillary, via the parenchymal tissue barrier. The tissue wall thickness (air-blood barrier thickness) is represented by δ, and the total septal wall thickness separating neighbouring alveoli is represented by *d*. (b) ^129^Xe MR spectra obtained from a healthy subject (black line) and a patient with IPF (blue line). (c) IDEAL CSI of dissolved ^129^Xe in the lungs of a patient with moderate COPD, illustrated in the form of ratio maps (reproduced with permission from [104]). (d) Representative binning maps and histograms derived from Dixon-based dissolved-phase ^129^Xe MRI acquired from a patient with IPF, highlighting the characteristic high TP (barrier) signal and low RBC signal compared with healthy normal subjects (dashed histogram), adapted with permission from [251]. (The notation barrier: gas is equivalent to TP/Gas.)

In a study demonstrating the clinical feasibility of ^129^Xe diffusion-weighted MRI [184], ^129^Xe ADC values obtained in COPD patients with emphysema (0.056 cm^2^/s) were significantly elevated in comparison to age-matched healthy controls (0.043 cm^2^/s). Other studies in COPD patients have reported global ^129^Xe ADC values ranging from 0.055 to 0.080 cm^2^/s [153], [157], [159], [185], [315], demonstrating the sensitivity of ^129^Xe ADC to emphysematous tissue destruction (shown histologically in Fig. 3a). In these studies, the ^129^Xe ADC value also significantly correlated with clinical measures of lung function, such as spirometry, the transfer factor of the lung for carbon monoxide (T_LCO_; a measure of gas exchange), and quantitative CT metrics of emphysema.

Further studies have demonstrated that ^129^Xe ADC is elevated, relative to younger healthy lungs, in older healthy volunteers [184], ex-smokers [157], [185], [188], and in patients with idiopathic pulmonary fibrosis [189] and lymphangioleiomyomatosis [168].

^129^Xe ADC measurements are highly repeatable in COPD patients [157], and demonstrate excellent linear correlation with ^3^He ADC across a range of microstructural length scales [157], [159], [185], [315], indicating that ^129^Xe ADC mapping is a robust methodology for imaging the lung microstructure. Importantly, ^129^Xe ADC acquired from explanted human [190] and animal [191], [192], [316] lungs are significantly correlated with histologically derived mean linear intercept (Lm), a widely accepted measure of airspace size. Whilst the ADC value is sensitive to changes in alveolar dimensions, the measured ADC is also dependent upon experimental acquisition parameters such as B_0_ field strength [193], and diffusion-encoding pulsed gradient strength, orientation and timing [194], [195], [196]. As such, it is difficult to directly link ADC measurements with lung morphometry parameters from histology, and to compare data between different sites that were acquired with different diffusion-weighting sequence parameters.

### 6.1 Theoretical models of hyperpolarised gas diffusion

The numerous airways of various sizes and orientations with respect to the diffusion-encoding pulsed gradient contained within each diffusion-weighted image voxel manifest as a non-Gaussian diffusion regime with a signal behaviour that deviates from the mono-exponential decay in Eq. (2) [197]. Theoretical models of hyperpolarised gas diffusion have been proposed to account for this non-Gaussian diffusion MR signal behaviour and extract measurements of acinar length scales. Much work has been performed in modelling the effects of restricted ^3^He and ^129^Xe diffusion using geometrical models of lung microstructure that include: cylindrical geometries [198], [199], [200], [201], acinar trees [202], branching structures [203], [204], [205], alveolar ducts [203], and porous media models [206], [207]. Alternative strategies have also been proposed that do not rely on geometrical assumptions of acinar structure: q-space transform analysis [208], [209], diffusion kurtosis [210] and stretched exponential models [95], [101], [211]. To date, cylindrical geometry [201], and stretched exponential [95] models are the only theoretical gas diffusion models that have been used to derive estimates of acinar length scales on a voxel-by-voxel basis from *in vivo* ^129^Xe diffusion MRI measurements, akin to those from obtained from histology.

The relatively small variation in acinar airway radii across the lungs [212] forms the foundation for a model of acinar airway geometry, known as the “cylinder” model [198], [200]. In this model, the acinar airways are modelled as infinitely long cylinders that are covered by alveolar sleeves containing eight alveoli and are characterised by two parameters, the outer acinar airway radii (R), and alveolar sleeve depth (h) (Fig. 3c). Underpinning this acinar airway geometry is a model of anisotropic diffusion, where more diffusion restriction exists perpendicular to the airway axis due to the alveolar walls, while less restriction is observed along the airway axis [198]. The derived longitudinal (*D_L_*) and transverse (*D_T_*) diffusion coefficients are related to the cylindrical airway geometrical parameters (*R* and *h*) by phenomenological expressions derived from Monte-Carlo simulations [199], [200], [201]. Additional parameters such as the alveolar volume (*V_Alv_*), alveolar surface area (*S_Alv_*), and mean chord length (*Lm*) can then be derived based upon the underlying cylindrical airway geometry.

An alternative theoretical model of hyperpolarised gas diffusion signal behaviour in the lungs is the stretched exponential model, in which *in vivo* estimates of alveolar length scales are derived without making assumptions about the geometry of the lung microstructure [95], [101], [211]. In this model, the stretched exponential function is fitted to the measured macroscopic voxel signal attenuation that can be represented as a superposition of signals with different apparent diffusivities (*D*) arising from airways with different sizes and orientations [95]. A probability distribution of apparent diffusivities can be estimated from the stretched exponential function parameters [213] and subsequently related to diffusive length scales, representative of the distribution of microscopic dimensions of the acinar airways (i.e. the diffusion-restricting boundaries) contained within a given voxel. The shape of this distribution is comparable to that of intercept lengths measured by histology, and can be used to derive a mean diffusion length scale (*Lm_D_*) representative of mean acinar airway dimensions within a voxel (Fig. 3d).

The first *in vivo* measurements of ^129^Xe lung morphometry were measurements of R and Lm derived from the cylindrical model [201] in a healthy subject and a patient with cystic fibrosis [214]. The first patient studies of *in vivo* ^129^Xe lung morphometry measurements were presented in four healthy never-smokers and four ex-smokers with COPD [215], [216]. Cylindrical model anisotropic diffusion coefficients and morphological parameters *R* and *Lm* were significantly increased in COPD patients while *h* was reduced compared with the healthy group. Further patient studies using the stretched exponential model have demonstrated elevated ^129^Xe $Lm_{D}$, with respect to young healthy lungs, in the lungs of ex-smokers, and in patients with IPF and COPD [95], [189].

Excellent agreement between ^129^Xe and ^3^He lung morphometry measurements derived from the stretched exponential and cylindrical geometry model has been demonstrated across a range of acinar length scales [95]. However, the validation of ^129^Xe lung morphometry measurements derived from theoretical gas models against gold standard methods for morphometry measurement (namely, histology) has to date been restricted to preclinical studies. For example, ^129^Xe lung morphometry measurements from the cylindrical geometry model were compared to histology in healthy mice lungs [316], and in rat lungs instilled with disease models of emphysema and radiation-induced lung injury [217], [218], [219]. Strong correlations were observed between histologically derived mean linear intercept and cylindrical geometry parameters. Furthermore, good agreement (10–30 µm difference) was observed between ^129^Xe MR-derived *Lm* and mean linear intercept from histology [218], [316].

In conclusion, diffusion-weighted MR imaging with hyperpolarised ^129^Xe is a robust methodology that is sensitive to acinar airspace size changes expressed in terms of ADC values and *in vivo* lung morphometry measurements from theoretical gas diffusion models.

## 7 Probing gas exchange with dissolved-phase ^129^Xe lung MRI and MRS

The primary function of the lungs is to facilitate the exchange of gases between the alveolar airspace and pulmonary capillaries. However, there is currently a lack of robust, quantitative biomarkers for spatially resolved assessment of pathological gas exchange impairment, and with sensitivity to disease progression and response to treatment. Dissolved-phase ^129^Xe MRS/I – wherein ^129^Xe spins dissolved in the lung parenchyma and capillaries are detected during their diffusive exchange with ^129^Xe gas in the alveoli (Fig. 4a) – may offer a solution.

### 7.1 Spectroscopic methods

The first *in vivo* HP ^129^Xe MR lung spectra were acquired from rat and mouse lungs [220], [221], and revealed the existence of multiple dissolved-phase ^129^Xe resonances, distinct from the dominant resonance of gaseous ^129^Xe in the alveoli. Shortly afterwards, similar resonances were observed in the human pulmonary system [222], and with the aid of *in vitro* studies [108], [223], the two *in vivo* dissolved-phase ^129^Xe resonances – tissue and blood plasma (TP) at ~197 ppm and red blood cells (RBCs) at 216–222 ppm – were identified. Representative dissolved-phased ^129^Xe NMR spectra obtained from the lungs of a healthy volunteer and a patient with idiopathic pulmonary fibrosis (IPF) are shown in Fig. 4b.

Several means to quantify pulmonary gas exchange *in vivo* by MR spectroscopy with hyperpolarised ^129^Xe have been reported. In the chemical shift saturation-recovery (CSSR) experiment, the MR signal of dissolved-phase ^129^Xe is saturated with a selective 90° RF pulse and the subsequent signal increase due to exchange with alveolar gaseous HP ^129^Xe is recorded as a function of post-saturation delay by MR spectroscopy. It was first reported in canines that the dissolved-phase ^129^Xe signal as a function of delay time shows two distinct trends: (i) an initial exponential increase with a plateau at ~200 ms, due to the saturation of parenchymal tissue with fresh HP ^129^Xe signal (i.e. related to gas exchange); and (ii) an approximately linear increase after ~1 s [224] due to blood flow (i.e. related to perfusion) [225]. Xenon gas exchange can be modelled by considering two adjacent alveoli separated by a “slab” of parenchymal tissue and capillaries (see Fig. 4a) in order to derive metrics of pulmonary function, including alveolar surface area to volume ratio (S/V), parenchymal septal thickness and capillary blood flow [225], [226], [227], [228], [229]. The CSSR method has been applied to assess gas exchange impairment in small animal emphysema and human patients with COPD, revealing tissue destruction (reduced S/V) [230], [231] and alveolar septal wall thickening [188], [232]. In IPF patients, pronounced septal thickening has been observed, consistent with pulmonary fibrosis [106], [227]. Model-derived lung function metrics are sensitive to the lung inflation level [231], [232], [317], and the septal thickness correlates with the clinical standard pulmonary function test for gas exchange (T_LCO_) [106], [232] and was found to be repeatable in patients with COPD [231].

Steady-state spectroscopic measurements (i.e. acquired with a single delay time) also allow quantitative assessment of gas exchange. The ratio of the ^129^Xe resonances of RBC to TP in the lung is sensitive to thickening of the alveolar septae in IPF (Fig. 4b) and also correlates with T_LCO_ [233]. This approach exhibits longitudinal sensitivity to disease progression in IPF where T_LCO_ showed no change [234], which has implications for disease management. Recent efforts have been concentrated on characterisation of the dissolved-phase ^129^Xe resonance lineshapes [235], accurate chemical shift referencing [236] and investigating oscillations in the RBC signal (and chemical shift) which track the cardiac cycle [237], [238]; the latter is discussed further in Section 8.

### 7.2 Imaging methods

As pathological gas-exchange impairment is spatially heterogeneous, 3D spatial information is required for improved disease management and targeted treatment in diseases such as IPF. Simultaneous imaging of dissolved-phase and gaseous-phase ^129^Xe in the lung within a single read-out can be achieved by tuning to the dissolved ^129^Xe resonance and choosing a low imaging bandwidth to exploit the chemical shift “artefact” [109]. However, this approach is constrained by SNR and resolution, and use of an ultra-short echo time radial sequence with interleaved frequency switching between dissolved-phase and gaseous-phase ^129^Xe offers improved SNR performance in light of the short T_2_* [239], [240]. While these techniques enable imaging of dissolved phase ^129^Xe (TP and RBC), distinction of the two dissolved-phase ^129^Xe compartments is important for quantitative gas exchange measurements. Free induction decay (FID)-based chemical shift imaging (CSI) with Cartesian phase encoding allows the acquisition of spatially-resolved spectra [241], though it suffers in terms of speed and spatial resolution. Dixon-type acquisitions [242] can be used to separate the TP and RBC resonances by exploiting the phase difference between them. In particular, single-point Dixon imaging has been developed for ^129^Xe gas exchange imaging; radially-encoded images are acquired at a TE where the phase difference between TP and RBC resonances is 90° [243]. The resulting RBC image represents ^129^Xe that has fully traversed the lung tissue barrier. Compared with “multi-point” approaches, the single-point method suffers from contamination of dissolved-phase ^129^Xe images with gas-phase ^129^Xe signals, though a technique for removal of this contamination has been reported [244]. An alternative approach is based on iterative decomposition with echo asymmetric and least-squares estimation (IDEAL) [245], which involves acquisition of images at multiple echo times to improve the separation of gaseous, TP- and RBC-dissolved ^129^Xe [104]. Both radial [104], [318] and spiral [246], [247] read-outs have been reported. The single-point Dixon method is sensitive to regional gas-exchange impairment in IPF, and dissolved-phase ^129^Xe MRI biomarkers show agreement with T_LCO_ [248], [318]. Recent data demonstrate the sensitivity of dissolved-phase ^129^Xe MRI to IPF disease progression [249], and distinct gas-exchange features in asthmatics and COPD patients [104], [232] and a range of cardiopulmonary pathologies [250].

While IDEAL or Dixon images are typically presented as ratio maps of RBC/TP, RBC/Gas or TP/Gas signals (see Fig. 4c), recent efforts have been focussed on improving quantitative analysis techniques, such as binning to create graded colour signal ratio maps to facilitate clinical interpretation [248], [251] (see Fig. 4d). In addition, further exploration of the reproducibility of these techniques is likely to aid clinical dissemination [252], [253]. Recent reports demonstrating the regional assessment of CSSR-type gas-exchange dynamics [254], [255], and novel techniques for imaging the cardiogenic oscillations of the ^129^Xe RBC resonance [256], may pave the way to maximising the obtainable functional information about pulmonary gas exchange with hyperpolarised ^129^Xe.

As *direct* dissolved-phase ^129^Xe imaging techniques are hampered by low SNR and short T_2_*, SNR benefits may be obtained by *indirect* gas exchange imaging, i.e. by detection of gaseous-phase ^129^Xe. The xenon polarisation transfer contrast (XTC) method is one such indirect technique, and involves (i) gradient echo imaging of gaseous phase ^129^Xe; (ii) a series of inversion/saturation RF pulses centred on the dissolved ^129^Xe resonances (to weight the signal intensity according to gas exchange); and (iii) further acquisition of gaseous-phase ^129^Xe images [257], [258]. The regional gas depolarisation between the two images is related to the gas exchange, can be modelled in terms of lung tissue density and thickness [258], and correlates with histological measurements of alveolar septal volume [259]. Repeating the XTC acquisition at several inter-pulse delay times (multiple exchange time XTC (MXTC) [260], [261]) permits mapping of a characteristic gas exchange constant and has been applied to quantify tissue loss in COPD [261]. The main limitation of XTC is that the TP and RBC resonances cannot be separated, since their close chemical shift and rapid chemical exchange inhibit selective inversion.

## 8 ^129^Xe dissolved in human blood

### 8.1 ^129^Xe relaxation in human blood

**Fig. 5:**
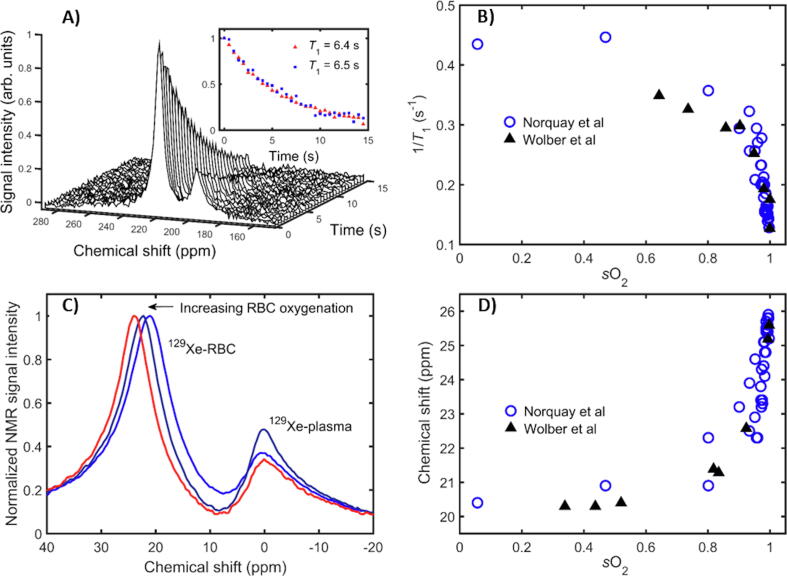
^129^Xe dissolved in human blood. (a) Decaying spectra from ^129^Xe dissolved in blood acquired with inter-pulse delay = 0.5 s. The inset shows a fit performed on the decreasing ^129^Xe NMR signal (integrals of ^129^Xe-red-blood-cell (RBC) and ^129^Xe-plasma absorption peaks) in order to establish ^129^Xe-RBC (red triangles) and ^129^Xe-plasma *T*_1_ values (blue squares). Here 0 ppm refers to the ^129^Xe gas-phase resonance frequency. The decaying spectra are from a blood sample with *s*O_2_ = 0.98. The data in (b) are the measured ^129^Xe relaxation rates (1/*T*_1_) in RBCs as a function of RBC oxygenation from [237] (open blue circles) and [264] (solid black triangles). In (c) it can be seen that with increasing oxygenation, the peak associated with ^129^Xe dissolved in RBCs is seen to shift measurably towards higher resonance frequency. Here 0 ppm is in reference to the ^129^Xe-plasma resonance frequency. Shown in (d) is a plot of the change in ^129^Xe-RBC chemical shift as a function of RBC oxygenation from Refs. [237] (open blue circles) and [264] (solid black triangles).

The ^129^Xe relaxation rate in blood has been studied in previous NMR experiments performed by several groups. In work conducted at a field strength of 4.7 T with hyperpolarised ^129^Xe [111]*,* the spin–lattice relaxation time, *T*_1_(^129^Xe), in red blood cells (RBC) within whole blood was found to increase with blood oxygenation (*s*O_2_), with *T*_1_(^129^Xe) values of 4 s and 13 s in deoxygenated and oxygenated blood, respectively. The same group also performed measurements with thermally polarised ^129^Xe samples and found the RBC *T*_1_(^129^Xe) in deoxygenated and oxygenated blood samples to be lower; 2.7 ± 0.22 s and 7.88 ± 0.16 s [262]. Work at a field strength of 1.5 T [223], also reported an increase in *T*_1_(^129^Xe) with blood oxygenation (2.88 ± 0.27 s deoxygenated and 5.71 ± 0.35 s oxygenated blood), and found the *R*_1_ = 1/ *T*_1_ to increase (or *T*_1_(^129^Xe) to decrease) non-linearly with blood oxygenation – see Fig. 5b. Both groups found the *T*_1_(^129^Xe) to be highest in blood that had been equilibrated with carbon monoxide, which locks the haemoglobin molecule into a conformation similar to fully oxygenated haemoglobin; Albert et al. [262] reported a value *T*_1_(^129^Xe) = 11 ± 2 s and Tseng et al. [223] reported a value *T*_1_(^129^Xe) = 7.84 ± 0.47 s.

In contrast, in a study conducted with a foam preparation of blood, at a field strength of 4.7 T [263], the opposite dependence of *T*_1_(^129^Xe) on blood oxygenation was observed. The *T*_1_(^129^Xe) was reported to decrease from 40 s in deoxygenated blood to 20 s in oxygenated blood, and it was deduced that interactions between xenon and paramagnetic bubbles of oxygen gas in the blood were the principal cause of spin-lattice relaxation. The interior of the bubbles provides a residency space for gaseous xenon and oxygen, and the bubble-walls provide a surface compartment for the oxygen-exposed gaseous xenon in which to dissolve. Xenon gas and paramagnetic oxygen gas in the bubbles (undergoing nuclear-electron dipole-dipole *T*_1_(^129^Xe) relaxation with a dependence inversely proportional to *p*O_2_) can readily exchange with the dissolved xenon in this regime and as such, the effect of oxygen on *T*_1_(^129^Xe) may have been overestimated.

Recently, RBC *T*_1_(^129^Xe) was examined over the widest yet range of blood oxygenations (*s*O_2_ values of 0.06–1.00) using hyperpolarised ^129^Xe [113] at a field strength of 1.5 T, where it was found that *T*_1_ increases non-linearly with blood oxygenation [see Fig. 5b], in agreement with the previous findings of Ref. [223]. In addition, the authors in Ref. [113] developed a two-site (RBCs and plasma) exchange model of the magnetization dynamics of ^129^Xe in whole blood samples to determine 10 constants that underpin and describe ^129^Xe NMR relaxation and exchange in isolated RBCs and isolated plasma, as well as in whole blood samples. Four constants were extrapolated by fitting the equation $1/T_{1}=\kappa \left[1-\exp{(r}_{sO_{2}}sO_{2})\right]+1/T_{1}^{dHb}$ to the blue circle data in Fig. 5b. Here $\kappa =4.6\times 10^{-6}$ s^−1^ is a scaling constant, $r_{sO_{2}}$= 11.1, is a “relaxivity index” characterizing the rate of change of ^129^Xe relaxation as a function of blood oxygenation and $1/T_{1}^{oHb}$ = 0.13 s^−1^ and$1/T_{1}^{dHb}$ = 0.42 s^−1^ are the ^129^Xe relaxation rates in fully oxygenated blood and fully deoxygenated blood, respectively. Two rate constants, *k*_a_ = 0.022 ms^−1^ and *k*_b_ = 0.062 ms^−1^, were determined for xenon diffusing between RBCs and plasma, respectively and two constants describing ^129^Xe relaxation within isolated plasma samples were determined:$r_{sO_{2}}$ = 0.075 s^−1^ mM^−1^ is the relaxivity index of ^129^Xe in the presence of dissolved molecular O_2_ within plasma and$1/T_{1,b}^{0}$ = 0.046 s^−1^ is the ^129^Xe relaxation rate in the absence of dissolved O_2_. The final two constants determined from the model represent the intrinsic ^129^Xe-RBC relaxation rates, $1/T_{1,a}^{oHb}$ = 0.19 s^−1^ and$1/T_{1,a}^{dHb}$ = 0.84 s^−1^, in oxygenated blood and deoxygenated blood, respectively.

Knowledge of these constants is important for future experiments involving modelling of the signal dynamics of ^129^Xe as it travels in the blood from lungs to distal tissues such as the brain and kidneys.

### 8.2 ^129^Xe chemical shift in human blood

Studies at a field strength of 1.5 T have shown that the ^129^Xe chemical shift in RBCs within whole blood increases non-linearly with RBC blood oxygenation [237], [264] (see Fig. 5c and d) in a similar manner to the ^129^Xe relaxation dependence on blood oxygenation discussed above. The ^129^Xe chemical shift in plasma remains fixed in frequency over the whole blood oxygenation range (see Fig. 5c). Using the ^129^Xe-plasma resonance as a 0 ppm reference, the ^129^Xe-RBC chemical shift was observed to increase from 20.5 ppm in fully deoxygenated blood to 26 ppm in fully oxygenated blood (Fig. 5d). This observed chemical shift vs. oxygenation behaviour is consistent when the same experiment is performed at magnetic field strengths of 1.5 T and 3 T [237], indicating that it is a field-strength-independent effect.

Knowledge of tissue oxygenation can provide insight into the pathophysiology of a variety of diseases, e.g. in the discrimination of the penumbra following stroke [265] and identification of ischemia following myocardial infarction [266]. In lung diseases such as asthma and COPD, hypoxia can influence the lifetime and the functionality of neutrophils that are associated with inflammation in the lungs [267]. The demonstrated sensitivity of the ^129^Xe-RBC chemical shift to blood oxygenation [237], [264] is promising as it could be used to non-invasively probe tissue oxygenation within the lungs and well-perfused tissues distal to the lungs. ^129^Xe-RBC resonance shifts with lung oxygenation have been reported *in vivo* in healthy volunteers during breath hold [268], and in patients with IPF [233] where a decrease in the ^129^Xe-RBC resonance frequency (indicating lower oxygenation) was observed.

Norquay et al*.* [237] used the *in vitro* data of the ^129^Xe-RBC chemical shift dependence on *s*O_2_ (Fig. 5d) as a calibration curve to determine absolute oxygenation changes in the alveolar capillary bed during a breath-hold challenge undertaken by healthy volunteers. For a TR of 800 ms (of the order of the RBC alveolar capillary transit time), it was observed that the ^129^Xe-RBC chemical shift exhibited a periodic modulation at the same frequency as the ^129^Xe-RBC signal oscillation, and with a 180° phase difference. Using the *in vitro* calibration data, the *s*O_2_ in two healthy volunteers at the start of the breath-hold was measured to be ~0.87, dropping to ~0.80 after 35 s of breath-hold apnea. The experiment was repeated for a shorter TR of 100 ms, where both the ^129^Xe-RBC and ^129^Xe-TP signals were observed to oscillate close to the cardiac pulsation frequency, in agreement with ^129^Xe-RBC and ^129^Xe-TP signal oscillations observed in Refs. [269], [270]. (Lower frequency oscillations observed at TR = 800 ms are likely an alias of the higher frequency oscillation observed for TR = 100 ms.) It was concluded that the observed signal and chemical shift oscillations could be attributed to changes in blood flux/oxygenation in the capillaries during the cardiac cycle. These ^129^Xe-RBC cardiogenic oscillations have recently been observed in patients with COPD, IPF, left heart failure (LHF), and pulmonary arterial hypertension (PAH) [238], [250]. It was found that IPF patients exhibited increased RBC amplitude and shift oscillations compared to healthy volunteers. Patients with COPD and PAH both exhibited decreased RBC amplitude oscillations compared to healthy volunteers, and interestingly LHF was distinguishable from PAH by enhanced RBC amplitude oscillations. Thus, ^129^Xe-RBC cardiogenic oscillation measurements hold promise for the distinction of functional characteristics of different cardiopulmonary diseases.

## 9 Imaging inhaled hyperpolarised ^129^Xe beyond the lungs

The blood supply for the brain, kidney, skeletal muscle, liver and gastrointestinal system accounts for 80% of the cardiac output [271]. Among these distal organs, the brain and kidney have short arterial delivery times of ~4 s [272] and ~2 s respectively from the lungs, short enough for inhaled HP ^129^Xe to retain polarisation until delivery, enabling direct MR imaging [60], [63], [273], [274].

**Fig. 6:**
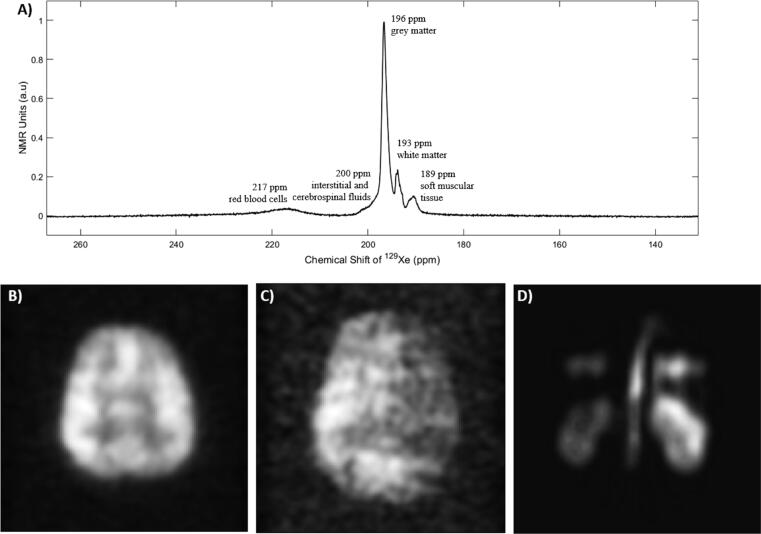
^129^Xe beyond the lungs. (a) Spectrum of HP ^129^Xe dissolved in the human head. Spectral peaks: 189 ppm: soft muscular tissue, 193 ppm: white matter, 196 ppm: grey matter, 200 ppm: interstitial and cerebrospinal fluids, and 217 ppm red blood cells. Brain perfusion images of (b) a healthy volunteer (adapted with permission from [60]) and (c) a volunteer with established stroke (adapted with permission from [281]), using inhaled hyperpolarised ^129^Xe. (d) Kidney perfusion images of a healthy volunteer using inhaled hyperpolarized ^129^Xe, adapted with permission from [63].

^129^Xe dissolved in the head *in vivo* exhibits five distinct NMR spectral peaks corresponding to: grey matter (196 ppm), white matter (193 ppm), interstitial and cerebrospinal fluids (200 ppm), soft muscular tissue (188 ppm) and red-blood cells (216 ppm) [11], [62], [63], [114], [275], [276], as seen in Fig. 6a. The NMR spectral peak from ^129^Xe dissolved in the grey matter dominates the spectrum [62], [114]. With a cerebral blood flow for grey matter of 65 mL per minute per 100 g of tissue [277], cerebral blood volume of 5 mL per 100 g of tissue [277] and Ostwald’s grey matter to blood partition coefficient of 0.88 [278] in healthy normal individuals, the inhaled HP ^129^Xe dissolved in the cerebral blood rapidly infuses in to the grey matter tissue reaching a concentration that enables direct MR imaging over a breath-hold of 24 s [60]. The MR image that is obtained (Fig. 6b) is a map of uptake of inhaled gas into the brain tissue across the intact blood-brain barrier, which is indicative of underlying physiology such as the regional cerebral blood flow and volume, regional mean transit time and gas transfer rate across the blood-brain barrier [60], [319]. Pre-clinical studies in rat brains have demonstrated image contrast sensitive to sensory stimuli [279] and induced ischemia [280]. Recently, there has been growing interest in the potential clinical sensitivity of inhaled HP ^129^Xe to human brain pathology such as Alzheimer’s disease [273] and stroke [281], as shown in Fig. 6c. HP ^129^Xe brain MRI benefits from the fact that inhaled ^129^Xe is safe and non-invasive, and crosses the intact blood-brain barrier, when compared to routine clinical CT and MR imaging techniques which use injected iodine- and gadolinium-based contrast agents respectively, both of which lead to a concern for patient safety [282], [283], [284]. In contrast to arterial spin labelling ^1^H MR imaging, HP ^129^Xe brain MRI does not require averaging [285], has no undesired signal from the intra-vascular compartment [60] and the image contrast directly depends on the underlying physiology.

In the kidneys, up to 3 spectral peaks have been demonstrated *in vivo*, two of which have been assigned to red blood cells (217 ppm) and tissue (and plasma) (198 ppm), while the origin of the third peak at 192 ppm is yet to be determined [274], [276], [286], [287]. A fourth peak at ~188 ppm is believed to originate from fat tissue in the lower abdomen, outside of the kidneys. With renal perfusion of 170 to 220 mL per minute per 100 mL of tissue [288], [289], the HP ^129^Xe saturates in the extra-vascular kidney compartment more quickly than in the brain, although with much lower spin density due to more rapid clearance of blood from the kidney. Nevertheless, imaging HP ^129^Xe dissolved in the kidney benefits from shorter arterial transit time, thereby less loss of polarisation due to T_1_ decay in blood prior to delivery, and a fast time-resolved acquisition can be used to characterise signal dynamics [286], [287]. Recent studies demonstrating MR spectroscopy and imaging of human kidneys *in vivo* using inhaled HP ^129^Xe [274], [286], [287] are encouraging, as shown in Fig. 6d.

## 10 Conclusions and future perspectives

^129^Xe lung MR is a versatile tool for the examination of lung function and structure, providing quantitative physiological information. Ventilation and diffusion MRI are well-established, repeatable techniques that are sensitive to early-stage lung disease [157], [167], [168], [169], [185], [188]. They have the potential to detect lung abnormalities earlier than spirometry [168], [169] and structural imaging [167], [290], allowing early intervention/therapy to mitigate further damage. Moreover, the sensitivity of ^129^Xe ventilation imaging to therapy response [158], [163], [171], [172] coupled with its high repeatability should enable clinical trials of novel therapeutics with small patient numbers, as has been demonstrated with ^3^He ventilation MRI [291], [292]. Crucially, the lack of ionising radiation associated with ^129^Xe MR is important when considering repeated imaging, and allows safe longitudinal monitoring of disease progression and studies of therapeutic response. This and the functional sensitivity of ^129^Xe MRI are real advantages in comparison to CT, the clinical gold standard for lung imaging. Nevertheless, the complementary use of ^129^Xe MRI alongside CT and/or advanced ^1^H morphological MRI allows the investigation of structure-function relationships to gain insights into disease pathology [167], [168], [293], [294].

**Fig. 7:**
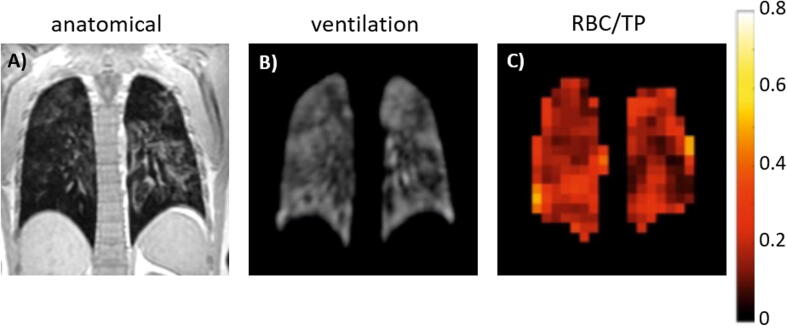
^129^Xe MRI in acute COVID-19. (a) ^1^H anatomical SPGR image, (b) ^129^Xe ventilation image and (c) ^129^Xe RBC/TP map acquired with an IDEAL sequence [318] of a coronal mid-posterior slice in a patient with acute COVID-19. For this slice, ventilated volume percentage = 99.6% and RBC/TP = 0.188 (mean RBC/TP in healthy volunteers = 0.47 [318]). RBC/TP = red blood cells to tissue and blood plasma ratio.

Dissolved-phase ^129^Xe MRS/I of the lung is still at an earlier stage of technological development, yet shows great promise as a probe for gas exchange. Increased signal from parenchymal lung tissue and reduced RBC signal indicate interstitial alveolar wall thickening and impaired gas transfer efficiency, and dissolved-phase ^129^Xe techniques have demonstrated the sensitivity to distinguish healthy smokers from never-smokers [188] and to detect longitudinal disease progression in patients with IPF earlier than currently used clinical metrics [234], [249]. During the course of the current COVID-19 pandemic, evidence is emerging that dissolved-phase ^129^Xe lung MRI is sensitive to gas exchange abnormalities in patients with COVID-19 [320]. The first published ^129^Xe MRI study in COVID-19 patients [320], conducted after patients had been discharged from hospital, found reduced gas-blood exchange function and lung ventilation with normal alveolar dimensions, while CT images showed substantial recovery compared to the peak stage of COVID-19.  Two patients scanned in the acute, symptomatic phase of COVID-19 at the University of Sheffield showed massively impaired gas exchange despite relatively normal ventilation (Fig. 7), and initial results from a collaboration between Oxford and Sheffield Universities indicate that gas exchange impairment is detectable using ^129^Xe MRI in patients three months after being ill with COVID-19 (bbc.co.uk/news/health-55017301).  Taken together, these initial findings suggest that dissolved-phase ^129^Xe lung MRI may provide a valuable tool for the investigation of COVID-19 lung disease. The concept of a single 3D gas- and dissolved- phase ^129^Xe MRI acquisition for regional assessment of gas exchange impairment and ventilation is attractive [192], [250], [295], [296], [297], although currently the gas-phase images obtained from multi-resonant imaging and dedicated ventilation images acquired separately are not interchangeable [297]. Further development of time-efficient, repeatable gas- and dissolved- phase imaging strategies with improved SNR may permit this in the future. In addition, the emergence of dissolved-phase ^129^Xe spectroscopy as a means to investigate blood oxygenation *in vivo* [233], [237], [264] and detect modulations of ^129^Xe RBC signal and chemical shift caused by the cardiac cycle [237], [270] that differ between patients with different cardiopulmonary disease types [238], [250] is of great clinical interest. Furthermore, evidence of reduced gas uptake following stroke [281] and retention of inhaled ^129^Xe in the brain in Alzheimer’s disease [273] measured by dissolved-phase ^129^Xe brain MRI are attractive to the neuroimaging community. It is likely that the multi-faceted potential of dissolved-phase ^129^Xe to probe oxygenation and gas exchange processes in the lungs, as well as perfusion in the brain and kidneys, will drive innovation in the field of hyperpolarised ^129^Xe MR in the years to come.

With current polarisation technologies, dedicated RF coil design and MR pulse sequence optimisation, high quality data acquisition with progressively lower doses of enriched xenon (86% ^129^Xe) and even natural abundance xenon (26% ^129^Xe) [78] has been made feasible, paving the way towards low cost ^129^Xe MRI. Following UK Medicine and Healthcare Regulatory Authority approval, routine clinical lung imaging has been performed in Sheffield, UK since 2015 [15], a service that has switched almost entirely to ^129^Xe in recent years [298]. This important milestone establishes routine clinical ^129^Xe lung MRI, opening the door to large-scale clinical evaluation of these methods in patient populations. In parallel, phase III clinical trials are currently in progress in the U.S. to obtain Food and Drug Administration approval for hyperpolarised ^129^Xe as a drug-device imaging agent [299], which should help drive international clinical trials.

There is a drive towards standardisation of ^129^Xe MR acquisition and analysis, to enable multi-site studies of ventilation imaging in the first instance (cpir.cchmc.org/XeMRICTC). A thermally polarised xenon torso phantom for quality assurance has been developed and tested at eight sites across North America [299]. Different approaches to image segmentation for ventilated volumes have been compared [152], [154], and substantial inter-reader agreement reported between blinded radiologists [164]. A recent retrospective study of ventilation images acquired at two institutions in children with CF found similar ventilation metrics between sites and strong agreement between two analysts, concluding that multi-centre trials in CF appear to be feasible [166]. Yet, there remains work to be done to standardise image acquisition and analysis between centres, each of which currently has its own established workflow tuned to their situation and preferences. This is critical not only for ventilation imaging, which is most well-developed and closest to clinical translation of the ^129^Xe MRI techniques, but also for diffusion-weighted ^129^Xe MRI and up-and-coming techniques such as dissolved-phase ^129^Xe MRS/I, to facilitate multi-site trials and aid their eventual transition to the clinic.

## Declaration of Competing Interest

The authors declare that they have no known competing financial interests or personal relationships that could have appeared to influence the work reported in this paper.

## Glossary

ADC: apparent diffusion coefficient
B_0_: main magnetic field
B_1_+: transmit magnetic field
CF: cystic fibrosis
CF-SEOP: continuous flow spin exchange optical pumping
COPD: chronic obstructive pulmonary disease
CSI: chemical shift imaging
CSSR: chemical shift saturation recovery
CT: computed tomography
D: diffusivity
DDC: distributed diffusivity coefficient
D_L_: longitudinal diffusion coefficient
D_0_: free diffusion coefficient
D_T_: transverse diffusion coefficient
FID: free induction decay
FLORET: Fermat Looped ORthogonally Encoded Trajectories
FRC: functional residual capacity
FVC: forced vital capacity
h: alveolar sleeve depth
^1^H: proton
^3^He: helium-3
HP: hyperpolarised
IDEAL: iterative decomposition with echo asymmetric and least-squares estimation
ILD: interstitial lung disease
IPF: idiopathic pulmonary fibrosis
LHF: left heart failure
Lm: mean linear intercept
Lm_D_: mean diffusion length scale
MR: magnetic resonance
MRI: magnetic resonance imaging
MRS: magnetic resonance spectroscopy
MXTC: multiple exchange time xenon polarisation transfer contrast
N_2_: nitrogen
NMR: nuclear magnetic resonance
PAH: pulmonary arterial hypertension
pO_2_: partial pressure of oxygen
Q_coil_: coil quality factor
R: outer acinar airway radii
Rb: rubidium
RBC: red blood cell
RF: radiofrequency
S_Alv_: alveolar surface area
SEOP: spin exchange optical pumping
SF-SEOP: stopped flow spin exchange optical pumping
SNR: signal to noise ratio
sO_2_: blood oxygenation
SPECT: single photon emission tomography
SPGR: spoiled gradient echo
S/V: surface area to volume ratio
T_1_: longitudinal relaxation time
T_2*_: transverse relaxation time
TLC: total lung capacity
T_LCO_: transfer coefficient for carbon monoxide
TP: tissue-plasma
TR: repetition time
UTE: ultra short echo time
V_Alv_: alveolar volume
VDP: ventilation defect percentage
VV%: ventilated volume percentage
ω: resonant frequency
^129^Xe: xenon-129
XTC: xenon polarisation transfer contrast

## Footnotes

1 Energy is transferred into the rotational/vibrational levels of the N_2_ molecules, resulting in non-radiative electronic decay.

2 Obstrctive lung diseases prevent patients from exhaling completely while restrictive lung diseases cause patients to have difficulty expanding their lungs fully.
